# Supplementary figures and images for: Dissecting Bayes: Using influence measures to test normative use of probability density information derived from a sample
Source: PLoS Comput Biol. 2024 May 1;20(5):e1011999. doi: 10.1371/journal.pcbi.1011999 (PMC11104641; doi:10.1371/journal.pcbi.1011999)

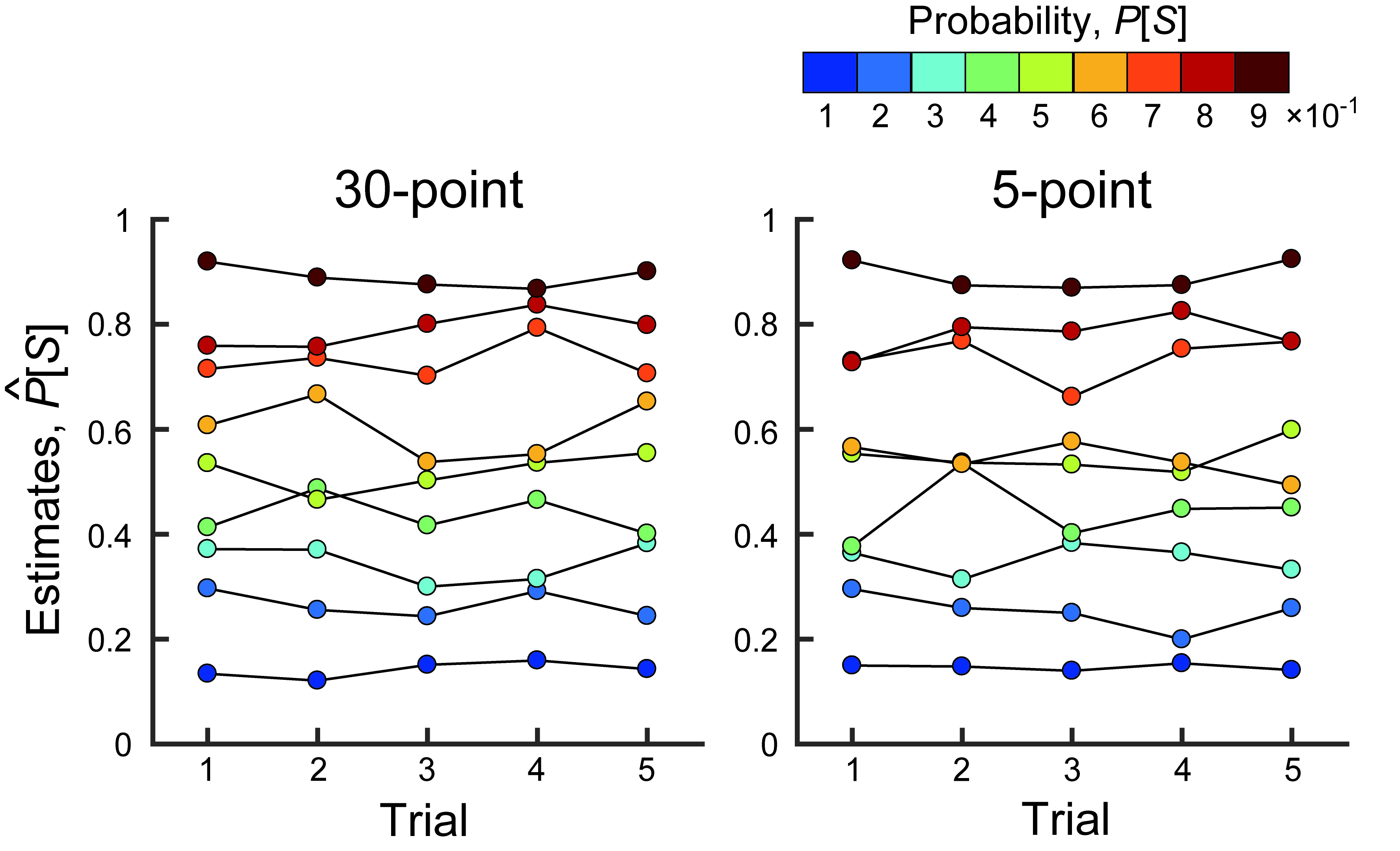

Supplement: S1 Fig — The participant’s estimates of probability in the symmetric interval are plotted versus trial. Data is averaged across the participants. The color scale of the circle indicates the correct probability between 0.1 and 0.9. The estimates were retained consistently from the beginning to the end of the task. Three-way within-participant ANOVA, using the correct probability (9), sample condition (2), and the number of trials (5) as independent variables, showed no significant main effect of the trial (F [2.6, 40.9] = 1.54, p = 0.22, η2 = 0.00). (TIF) [file pcbi.1011999.s001.tif]

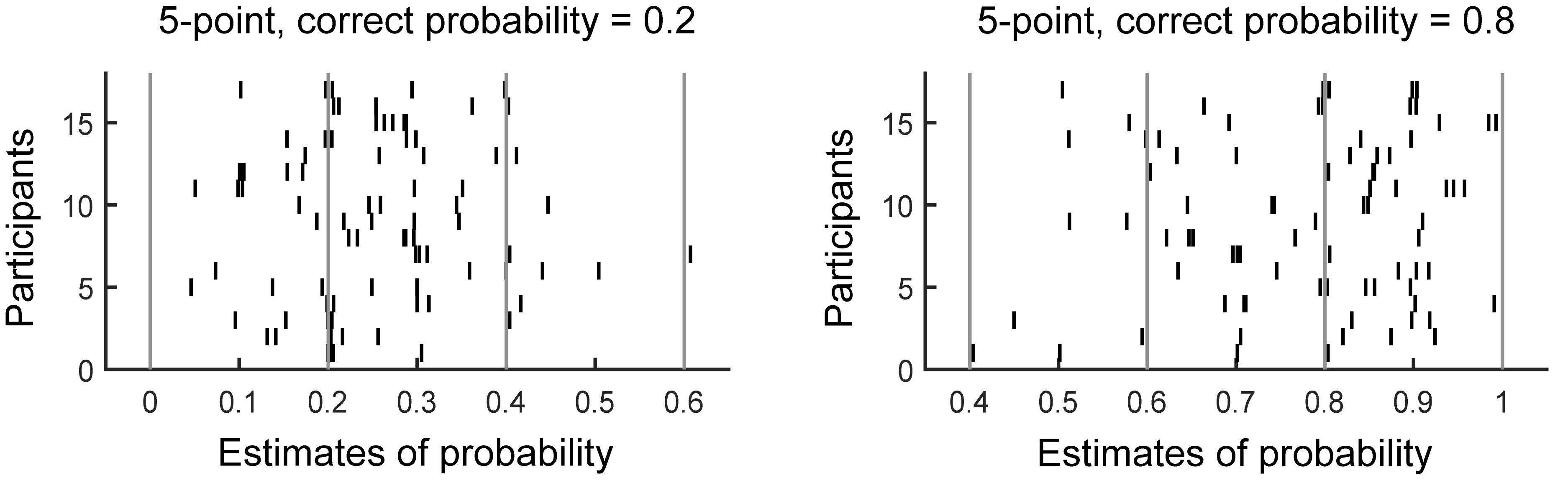

Supplement: S2 Fig — A small vertical line marks the observer’s estimate of probability in each trial (i.e., each sample). The data are taken from the symmetric interval condition. The vertical grey lines in lower panels indicate the possible probabilities taken by the counting point strategy. With a 5-point sample, these could be from 0.0 to 1.0 in steps of 0.2. For instance, the estimate would be 0.2 if one point falls within the interval. The counting strategy fails to predict the observers’ estimates of probability. (TIF) [file pcbi.1011999.s002.tif]

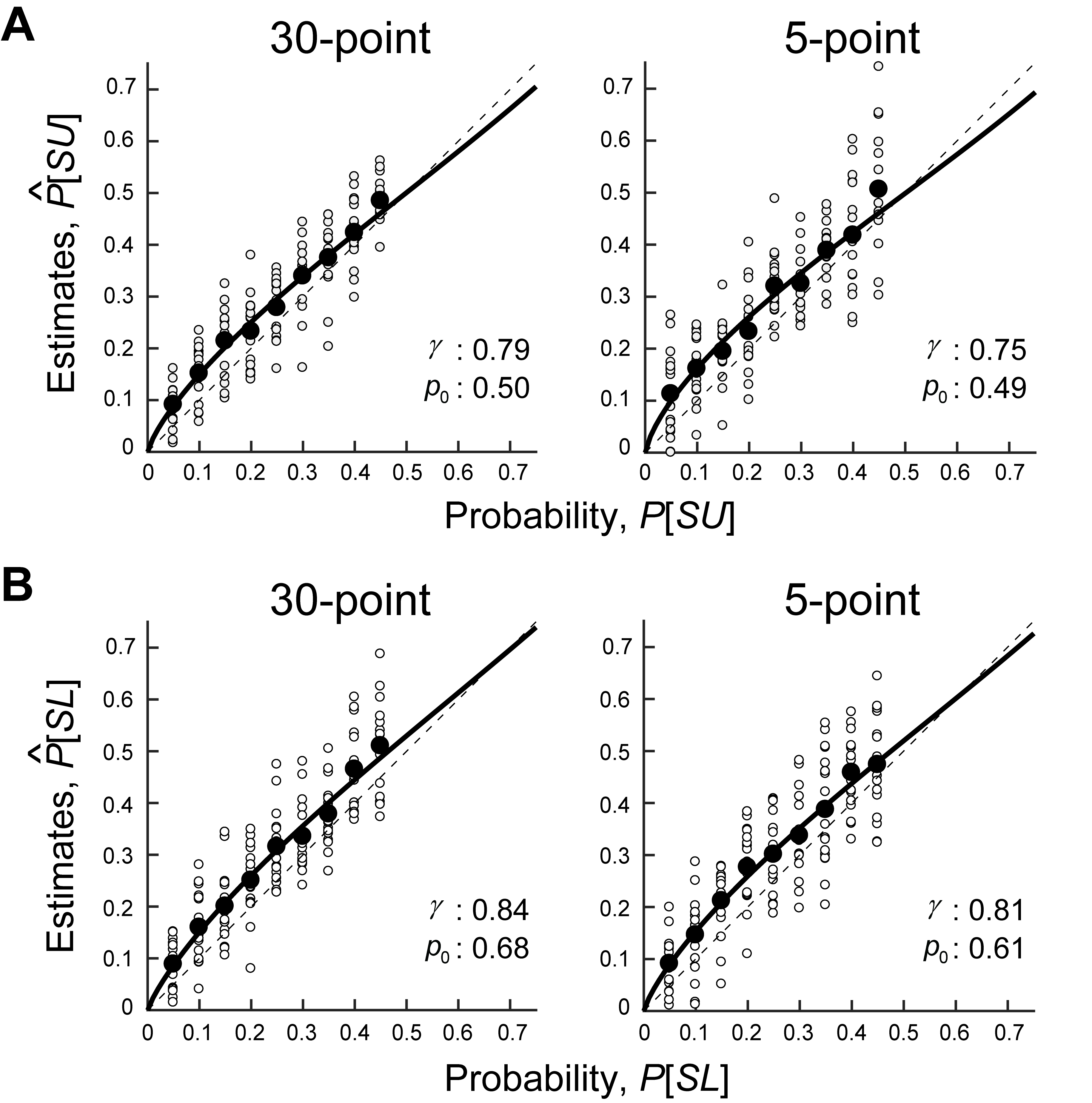

Supplement: S4 Fig — The participants’ estimates of probability in the upper half (A) and lower half (B) of the symmetric interval are plotted against the correct probability. Each white circle denotes the estimates for a single participant and a filled circle is the average estimates across participants. The black thick curve is the best-fit estimate by a linear in log-odds (LLO) function. The LLO parameters for each fit are γ and p0. (TIF) [file pcbi.1011999.s004.tif]

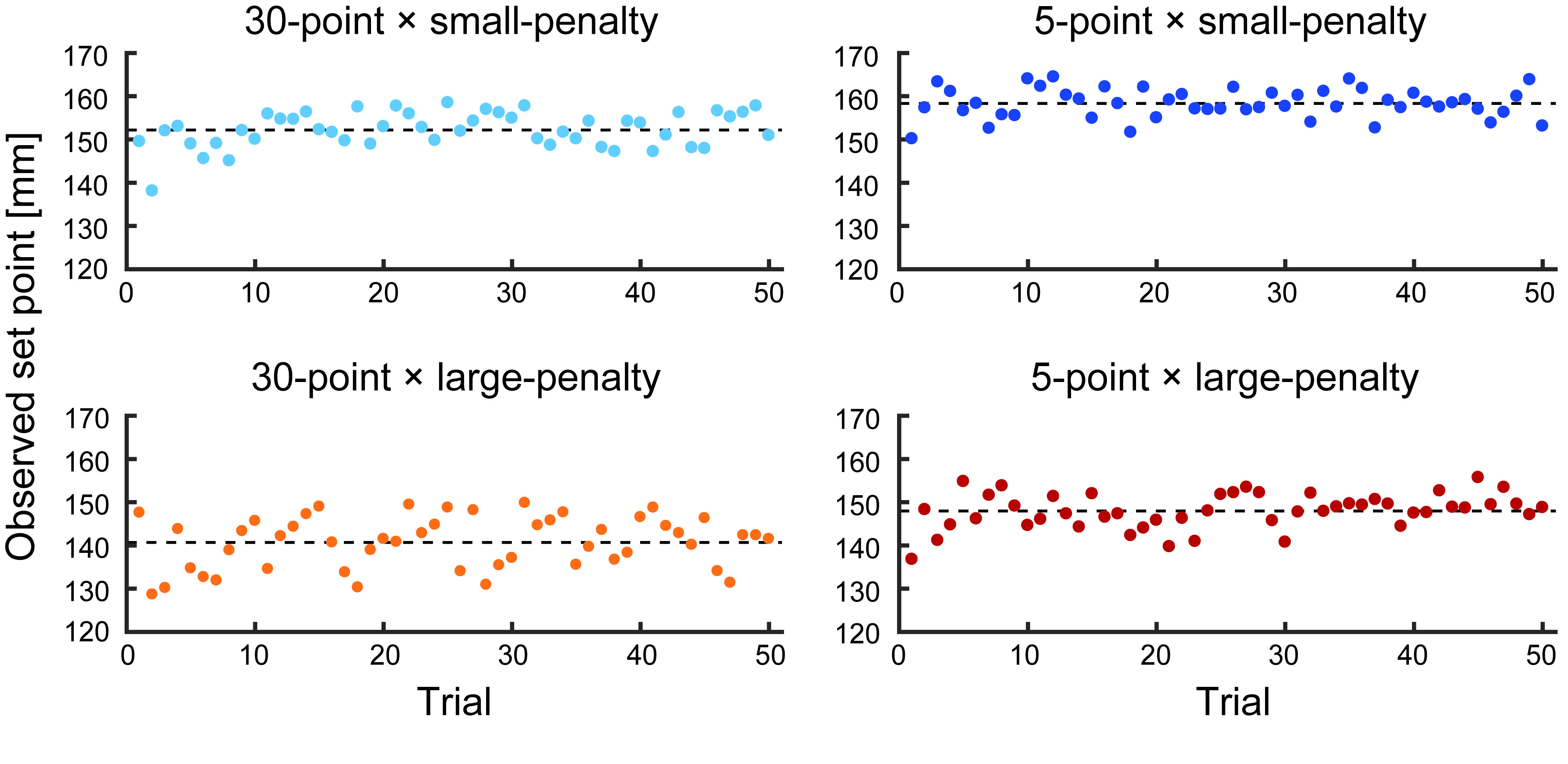

Supplement: S6 Fig — A trial-wise set point averaged over the participants is plotted for each condition. The horizontal dashed lines denote the mean set point across all trials. There is no evident pattern in the residuals. (TIF) [file pcbi.1011999.s006.tif]

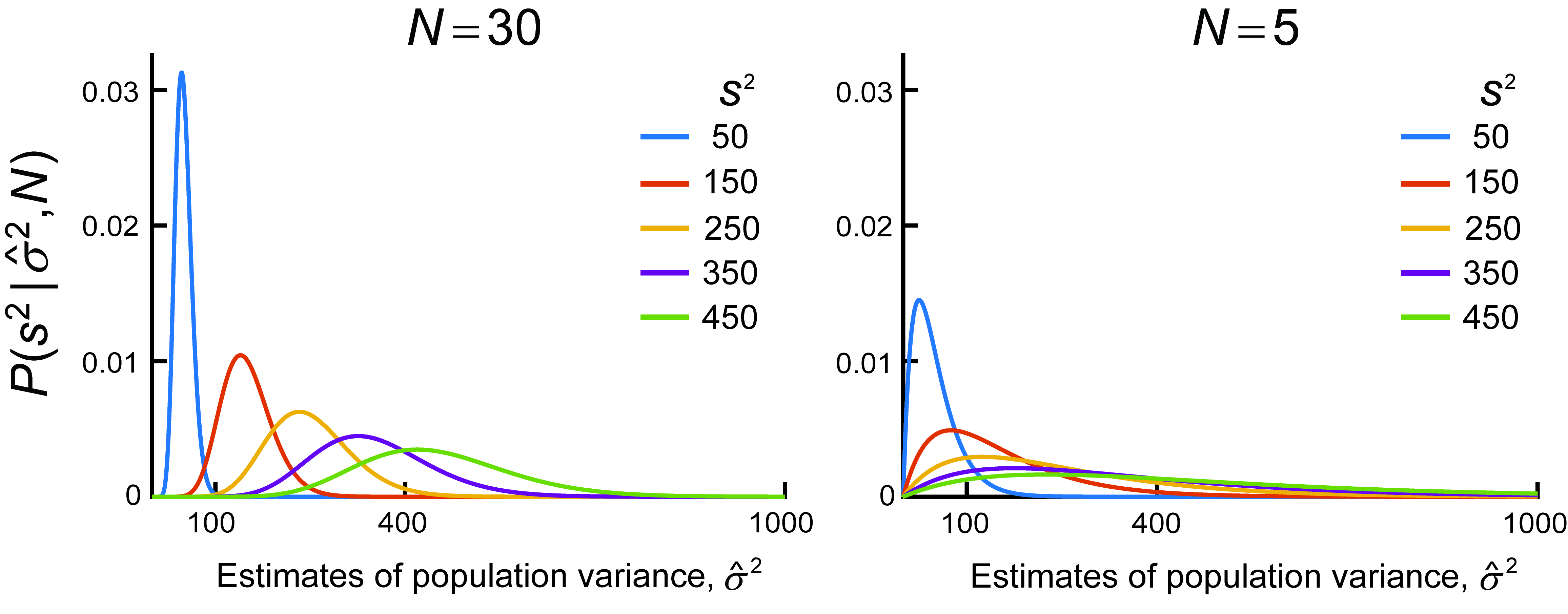

Supplement: S9 Fig — Given the population variance σ^2, the random variable of the sample variance s2 is distributed according to a chi-square distribution with N−1 degrees of freedom. Therefore, the likelihood function of the estimated population variance can be described as a chi-square probability density function. We show the likelihood functions when N = 30 (left) and N = 5 (right). The sample variance varied between 50 mm and 450 mm. (TIF) [file pcbi.1011999.s009.tif]

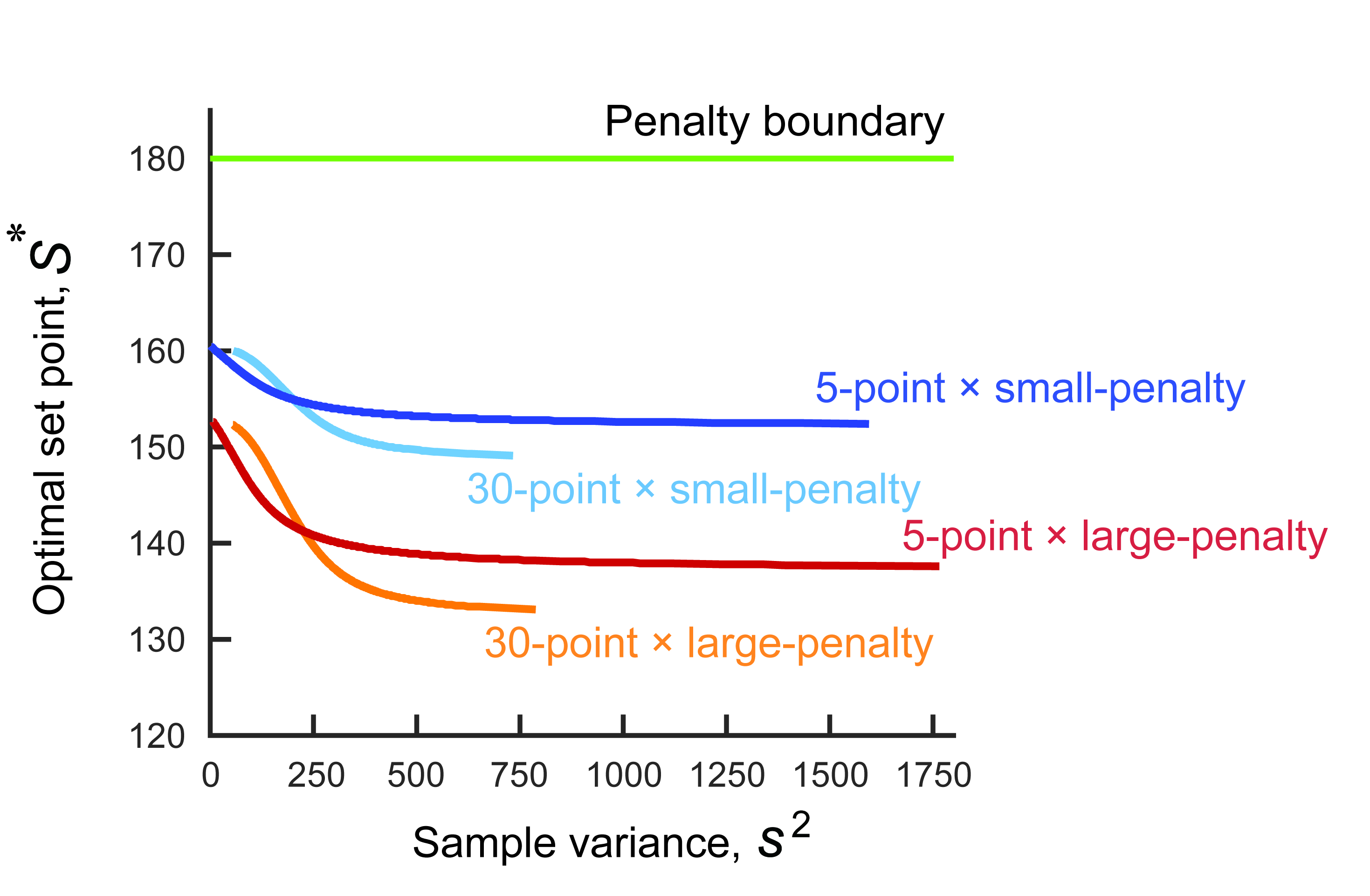

Supplement: S10 Fig — The optimal set point was modelled for the variance in the sample. The optimal set point changes from trial to trial as the sample points are resampled in each trial and the sample variance changes. In Fig 9C, the optimal set point is averaged across trials and is plotted against the observer’s average set point. (TIF) [file pcbi.1011999.s010.tif]
