# Supplementary material for: Dissecting Bayes: Using influence measures to test normative use of probability density information derived from a sample
Source: PLoS Comput Biol. 2024 May 1;20(5):e1011999. doi: 10.1371/journal.pcbi.1011999 (PMC11104641; doi:10.1371/journal.pcbi.1011999)
Supplement: S1 Text — Detalis of the model formula in the test of accuracy. (PDF) [file pcbi.1011999.s011.pdf]

S1 Text. Model comparison in the test of accuracy.

We fit participants' estimates in the interval estimation task to the two-parameter linear log-odds function [1] written as

$$\text{H1:} Lo(\widehat{P}[S]) = \gamma Lo(P[S]) + (1-\gamma)\, Lo(p_0) \quad \text{[S1-1]}$$

where $Lo(p) = \ln\left[p/(1-p)\right]$. The equation is written in terms of the symmetric interval *S* for convenience. It applies equally to *SU* and *SL* as well. In addition, we considered other hypotheses concerning the form of probability distortion

$$\text{H2:} \widehat{P}[S] = \frac{P[S]^{\gamma}}{\left(P[S]^{\gamma} + (1-P[S])^{\gamma}\right)^{1/\gamma}} \quad \text{[S1-2]}$$

$$\text{H3:} \widehat{P}[S] = \exp[-(-\text{Ln}(P[S]))^{\gamma}] \quad \text{[S1-3]}$$

where hypothesis H2 is a two-parameter version of the probability distortion function from [2]. H3 is the two-parameter version of the probability distortion function of [3]. We also compared the LLO function (H1) with a null hypothesis with perfect accuracy (no distortion).

$$\text{H0:} \widehat{P}[S] = P[S] \quad \text{[S1-4]}$$

We fit these models to the mean estimates across participants by maximum likelihood. Supporting Table 1 summarizes the results. An AIC model comparison indicates that the LLO function fits the data best.

References

1. Zhang H, Maloney LT. Ubiquitous log odds: a common representation of probability and frequency distortion in perception, action, and cognition.
2. Tversky A, Kahneman D. Advances in prospect theory: Cumulative representation of uncertainty Journal of Risk and uncertainty. 1992 5:297-323.
3. Prelec D. The Probability Weighting Function. Econometrica. 1998;66(3).
