# Supplementary material for: Dissecting Bayes: Using influence measures to test normative use of probability density information derived from a sample
Source: PLoS Comput Biol. 2024 May 1;20(5):e1011999. doi: 10.1371/journal.pcbi.1011999 (PMC11104641; doi:10.1371/journal.pcbi.1011999)
Supplement: S1 Table — Note—“No. Par.” is the number of free parameters for each model. ΔAICc = AICcH1−AICcHi. A positive AICc difference indicates a better-fitting model than H0 (no distortion). The evidence ratio is defined by exp(ΔiAICc2) and is the relative likelihood of model pairs and represents the evidence about models as to which is better in a K-L information sense. A value of evidence ratio means how many times the data is more likely under the alternative than the null hypothesis. Evidence ratios greater than 10 represent strong evidence for the alternative hypothesis whereas evidence ratios less than 0.1 represent strong evidence for the null hypothesis. Evidence ratios greater than 3 (or less than 0.33) represent substantial evidence for the alternative hypothesis (or for the null hypothesis). A value in the intermediate range (0.33 and 3) supports neither hypothesis. H1 (LLO function) is strongly supported. The right four columns summarize the free parameters that best describe the data: γ is the slope of the curve and p0 is the crossover point. (PDF) [file pcbi.1011999.s012.pdf]

S1 Table. Model comparison in the test of accuracy.

Condition	Model	No. Par.	Fit to the mean estimates				Recovered free parameter(s)			
			30	5	30	5	30	5	30	5
			Δi AICc		Evidence ratio		γ		p_0	
$P[S]$	H0	0	0.0	0.0	1.0	1.0				
	H1	2	12.9	1.5	629.2	2.2	0.88	0.87	0.76	0.72
	H2	1	0.1	0.0	1.07	1.0	0.92	0.90		
	H3	1	-2.1	-1.6	0.36	0.4	0.96	0.93		
$P[SU]$	H0	0	0.0	0.0	1.0	1.0				
	H1	2	14.9	8.8	1736	81.7	0.79	0.75	0.50	0.49
	H2	1	12.3	7.8	463	48.8	0.80	0.79		
	H3	1	6.4	4.5	25	9.6	0.77	0.74		
$P[SL]$	H0	0	0.0	0.0	1.0	1.0				
	H1	2	16.0	21.0	2977.1	36527.3	0.84	0.81	0.68	0.61
	H2	1	6.0	9.7	20.2	129.2	0.80	0.78		
	H3	1	2.1	4.8	2.9	11.1	0.76	0.74		
