# Supplementary material for: Dissecting Bayes: Using influence measures to test normative use of probability density information derived from a sample
Source: PLoS Comput Biol. 2024 May 1;20(5):e1011999. doi: 10.1371/journal.pcbi.1011999 (PMC11104641; doi:10.1371/journal.pcbi.1011999)
Supplement: S2 Table — See the note in S1 Table. ΔAICc = AICcH0−AICcHi. A positive AICc difference indicates a better-fitting model than a null hypothesis (H0). Three hypotheses concerning estimates for the test of additivity are below. A super-additive model (H1) outperformed the other models H0:P^[SU]+P^[SL]=P^[S] H1:P^[SU]+P^[SL]=P^[S]+b(b>0) H2:P^[SU]+P^[SL]=P^[S]+b(b<0). (PDF) [file pcbi.1011999.s013.pdf]

S2 Table. Model comparison in the test of additivity.

Model	No. Par.	Fit to the mean estimates				Recovered free parameter	
		30	5	30	5	30	5
		Δi AICc		Evidence ratio		b	
H0	0	0.0	0.0	1.0	1.0		
H1	1	14.8	18.3	1660	9336	0.063	0.072
H2	1	-2.6	-2.6	0.3	0.3	0.000	0.000
